# Nucleolar Dominance in a Tetraploidy Hybrid Lineage Derived From *Carassius auratus* red var. (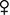) × *Megalobrama amblycephala* (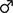)

**DOI:** 10.3389/fgene.2018.00386

**Published:** 2018-09-24

**Authors:** Liu Cao, QinBo Qin, Qiong Xiao, HongTing Yin, Jin Wen, QiWen Liu, Xu Huang, YangYang Huo, Min Tao, Chun Zhang, Kaikun Luo, ShaoJun Liu

**Affiliations:** ^1^State Key Laboratory of Developmental Biology of Freshwater Fish, Hunan Normal University, Changsha, China; ^2^College of Life Sciences, Hunan Normal University, Changsha, China

**Keywords:** nucleolar dominance, distant hybridization, polyploid hybrid progeny, *45S rRNA* gene, tetraploidization

## Abstract

Nucleolar dominance is related to the expression of *45S rRNA* genes inherited from one progenitor due to the silencing of the other progenitor’s *rRNA* genes. To investigate nucleolar dominance associated with tetraploidization, we analyzed the changes regarding the genetic traits and expression of *45S rRNA* genes in tetraploidy hybrid lineage including F_1_ allotetraploids (4n = 148) and F_2_ autotetraploids (4n = 200) derived from the distant hybridization of *Carassius auratus* red var. (2n = 100) (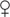) ×*Megalobrama amblycephala* (2n = 48) (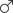). Results showed that nucleolar dominance from the females was established in F_1_ hybrids and it was inherited in F_2_ hybrids, suggesting that tetraploidization can lead to rapid establishment of nucleolar dominance in the hybrid origin’s tetraploid lineage. These results extend the knowledge of nucleolar dominance in polyploidy hybrid animals, which are of significance for the evolution of hybrids in vertebrates.

## Introduction

Polyploidy species have played a major role in the evolution and the adaptation of eukaryotes in both animals (Muller, 1925; Peer et al., 2009) and plants (Levy and Feldman, 2002; Soltis et al., 2009; Jiao et al., 2011). Hybridization is one of the primary mechanisms for the origin of species leading to the formation of polyploids (Otto and Whitton, 2000; Liu, 2010). In the newly formed allopolyploids, the genomes changes react in some genomic reorganizations and modifications of parental genomes. It is assumed that these genomic changes facilitate the establishment and success of the newly formed polyploids (Song et al., 1995; Ozkan et al., 2001; Kashkush et al., 2002; Raskina et al., 2002). In previous studies, fertile allotetraploids (abbreviated as F_1_) (AABB, 4n = 148) were successfully obtained in the first generation derived from the distant hybridization of *Carassius auratus* red var. (RCC) (RR, 2n = 100) (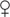) ×*Megalobrama amblycephala* (BSB) (BB, 2n = 48) (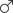) as a result of chromosome doubling of diploid hybrid embryos (AB, 2n = 74) (Liu et al., 2007). Fertile autotetraploids (abbreviated as F_2_) (AAAA, 4n = 200) were obtained in the second generation through crossing autodiploid sperm and autodiploid ova produced by abnormal chromosome behavior during meiosis of F_1_ hybrids (Qin et al., 2014). In contrast with the F_1_ hybrids, the F_2_ hybrids have increasing the fertility and reach sexual maturity at 1 year old.

By self-mating, F_2_ hybrids can generate the next generation of the autotetraploids. Until now, a stable autotetraploid line (F_2_ – F_13_; 4n = 200) is formed which can provides an excellent materials to investigate the mechanisms that drive diploidization in autotetraploids and is useful in the production of the sterile triploids (Qin et al., 2014). This hybrid origin’s tetraploid lineage is an attractive model to elucidate genomic changes associated with tetraploidization.

Nucleoli, the sites of *45S rRNA* gene transcription (the *5.8S*, *18S*, and *28S rRNA*) and ribosome assembly, form at the chromosome loci where tandemly repeated *45S rRNA* genes are actively transcribed (Reeder, 1985; Comai, 2000). In a number of interspecific hybrids, nucleolar dominance describes expression of *45S rRNA* genes inherited from one progenitor while silencing of the other progenitor’s *rRNA* genes (Tucker et al., 2010). About 70 years of research has contributed to our current understanding of nucleolar dominance, through studies based on plants, invertebrates, frogs, flies, fish, and mammals (Mcstay, 2006; Preuss and Pikaard, 2007). The transcriptional dominance of the ribosomal genes of one species over the ribosomal genes of another species was first described in some interspecific hybrids of *Crepis* (Honjo and Reeder, 1973), and was then confirmed by the studies of other hybrids or allopolyploid species (Lacadena et al., 1984; Reeder, 1985; Gustafson et al., 1988). At present, the knowledge of understanding the nucleolar dominance in polyploid animals is still limited, because the formation of distant polyploid hybrid lineages in animals is challenging in practice.

The genetics traits and expression of *45S rRNA* genes have been studied by detecting single nucleotide polymorphism (SNP) sites in the *18S rRNA* gene (Flowers and Burton, 2006; Xiao et al., 2016). Nucleotide sequence variation in the internal transcribed spacer *(ITS*) region of the *45S rRNA* genes has been the candidate of choice for species identification (Jobes and Thien, 1997; Hajibabaei et al., 2007; Son et al., 2009) and phylogenomic analyses (Yonemori et al., 2002; Liu et al., 2008). Here, we analyzed the genetic traits and expression of *45S rRNA* genes in RCC, BSB, F_1_ and F_2_ hybrids. The results showed that nucleolar dominance from the females was established in F_1_ hybrids and it was inherited in F_2_ hybrids, suggesting that tetraploidization can lead to rapid establishment of nucleolar dominance in the hybrid origin’s tetraploid lineage. These results extend the knowledge of nucleolar dominance in polyploidy hybrid animals, which are of significance for the evolution of hybrids in vertebrates.

## Materials and Methods

### Source of Samples

The allotetraploids (F_1_, AABB, 4n = 148) were obtained in the first generation of the distant hybridization of *Carassius auratus* red var. (RCC, AA, 2n = 100 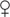) × *Megalobrama amblycephala* (BSB, BB, 2n = 48 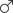) as a result of chromosome doubling of diploid hybrid embryos (AB, 2n = 74) (Liu et al., 2007). The autotetraploids (F_2_, AAAA, 4n = 200) were obtained in the second generation through crossing autodiploid sperm and autodiploid ova produced by abnormal chromosome behavior during meiosis of F_1_ hybrids (Qin et al., 2014). All samples were reared and bred at the Engineering Research Center of Polyploid Fish Breeding and Reproduction of the State Education Ministry, China, located at Hunan Normal University.

### Fluorescence *in situ* Hybridization (FISH)

To determine ploidy, a *5S* gene probe was made from RCC genome and amplified by PCR using the primers 5′-TTCGAAAAGAGAGAATAATCTA-3′ and 5′-AACTCGTCTAAACC CGAACTA-3′ (Qin et al., 2010). The fluorescence *in situ* hybridization (FISH) probes were produced by Dig-11-dUTP labeling (using a nick translation kit; Roche, Germany) of purified PCR products. FISH was performed according to He et al. (2012). Two hundred metaphase chromosome spreads from 10 individuals were analyzed for each type of fish (RCC, BSB, F_1_ and F_2_ hybrids). Preparations were examined under an inverted microscope (CW4000, Leica, Germany), with a confocal imaging system (LCS SP2, Leica). Captured images were colored and superimposed in Adobe Photoshop CS6.

### Analysis of the Genetics Traits and Expression in *ITS* Sequences

The *ITS* sequences from different genomes were amplified using *ITS* primers (5′-AGTCGTAACAAGGTTTCCGTAGGTG-3′; 3′-TTATGGCCGTGCTCTGGCTAT-5′), with DNA and cDNA as templates. This pair of primers was designed based on the *45S* sequence of common carp (GenBank, Accession No. JN628435.1). Unique cleavage sites for restriction enzymes (*Not*I, *Dra*I, *Stu*I; New England Biolabs) were located in *ITS* sequences from different genomes using a sequence alignment program (GeneTool) (Beisvag et al., 2006). Three individuals for each type of fish (RCC, BSB, F_1_ and F_2_ hybrids) were used to validate these restriction enzymes sites.

### Analysis of the *18S rRNA* Gene Sequence and Detection of *45S rRNA* Gene Expression

A pair of primers reported by Singh et al. (2009) (*18S*, 5′-TTGGTGACTCTCGATAACCTCGGGC-3′; *18S*, 5′-CCTTGTTACGACTTTTACTTCCTC-3′) was used to amplify the *18S rDNA* fragment. Genomic DNA and total RNA were simultaneously isolated from single adult fish. Thirty individuals of each fish (including RCC, BSB, F_1_ and F_2_ hybrids) were selected at random. DNA was extracted with a universal genomic DNA extraction kit (TaKaRa). The liver tissue of different individuals was used to isolate total RNA using a Total RNA Isolation System (Omega Bio-Tek, Norcross, GA, United States). RNA was treated with gDNA Eraser (TaKaRa) and first-strand cDNA was synthesized with Superscript III (Invitrogen) using an *18S rRNA* gene-specific primer (5′-CCTTGTTACGACTTTTACTTCCTC-3′). Then the *18S* F/P primer pair was used to amplify the cDNA fragment of *18S rDNA* by PCR. For all samples, PCR controls were used to detect the contamination of RNA with genomic DNA. Sequences were analyzed with BioEdit (Hall, 1999) and Clustal W (Thompson et al., 1994). To examine the SNP sites, we designed three pairs of primers according to 100 upstream and downstream nucleotides in the SNP sites (**Supplementary Table S1**). Except the forward primer in position 41 was designed according to 200 upstream nucleotides.

## Results

### Examination by Fluorescence *in situ* Hybridization (FISH)

**FIGURE 1:**
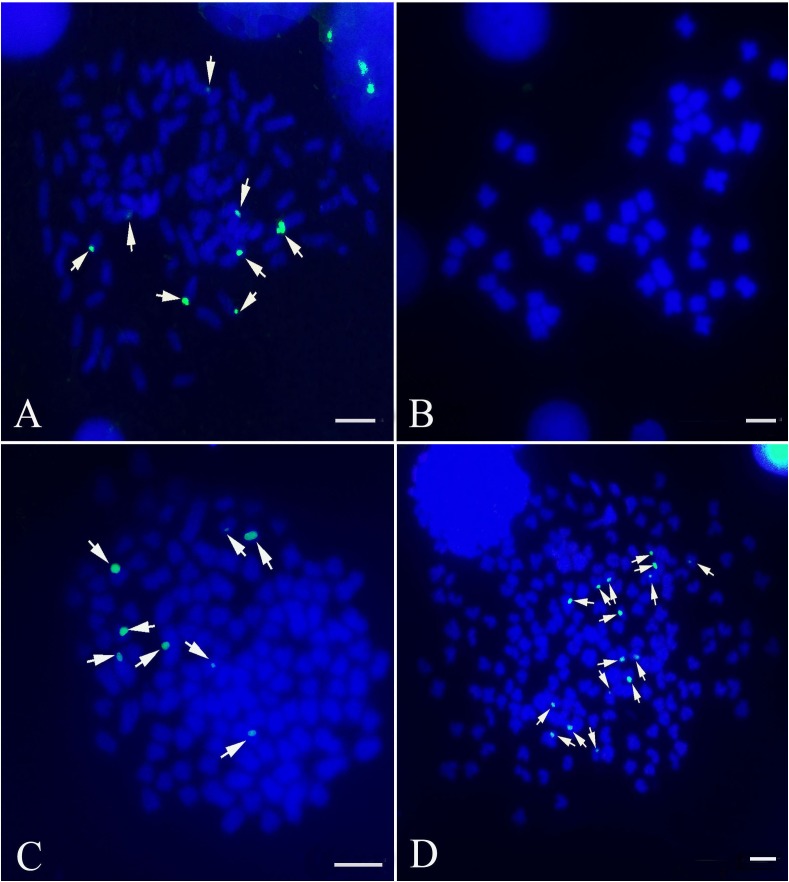
Examination of hybridizing signals by the *5S rDNA* probe. The white arrows indicate the *5S rDNA* gene loci. The eight *5S* gene loci were found in RCC **(A)**, but none in BSB **(B)**. The eight *5S* gene loci in F_1_ **(C)** and sixteen *5S* gene loci in F_2_ **(D)** are shown. Bars **(A–D)**: 3 μm. RCC, *Carassius auratus* red var.; BSB, *Megalobrama amblycephala*; F_1_, allotetraploids; F_2_, autotetraploids.

The *5S rDNA* probe (477 bp, GenBank: GQ485557) hybridized with the metaphase chromosomes of RCC, BSB, F_1_ and F_2_ hybrids. Hybridization of the *5S rDNA* probe yielded eight *5S* gene loci in RCC (**Figure 1A** and **Table 1**), but none in BSB (**Figure 1B** and **Table 1**); thus, RCC and BSB-derived chromosomes could be discriminated by the *5S rDNA* probe. In the F_1_ hybrids, eight RCC-derived *5S rDNA* gene loci were detected in the metaphase chromosomes (**Figure 1C** and **Table 1**). Sixteen RCC-derived *5S rDNA* gene loci were detected in the metaphase chromosomes of the F_2_ hybrids (**Figure 1D** and **Table 1**).

**Table 1:** Chromosomal locus numbers.

Fish type^a^	No. of fish	No. of metaphases	No. of *5S rDNA* loci
RCC	10	200	8
BSB	10	200	0
F_1_	10	200	8
F_2_	10	200	16

*^a^RCC, Carassius auratus red var.; BSB, Megalobrama amblycephala; F_1_, allotetraploids; F_2_, autotetraploids.*

### Inheritance Pattern of *ITS* Sequences in Parental Species and Hybrids

**FIGURE 2:**
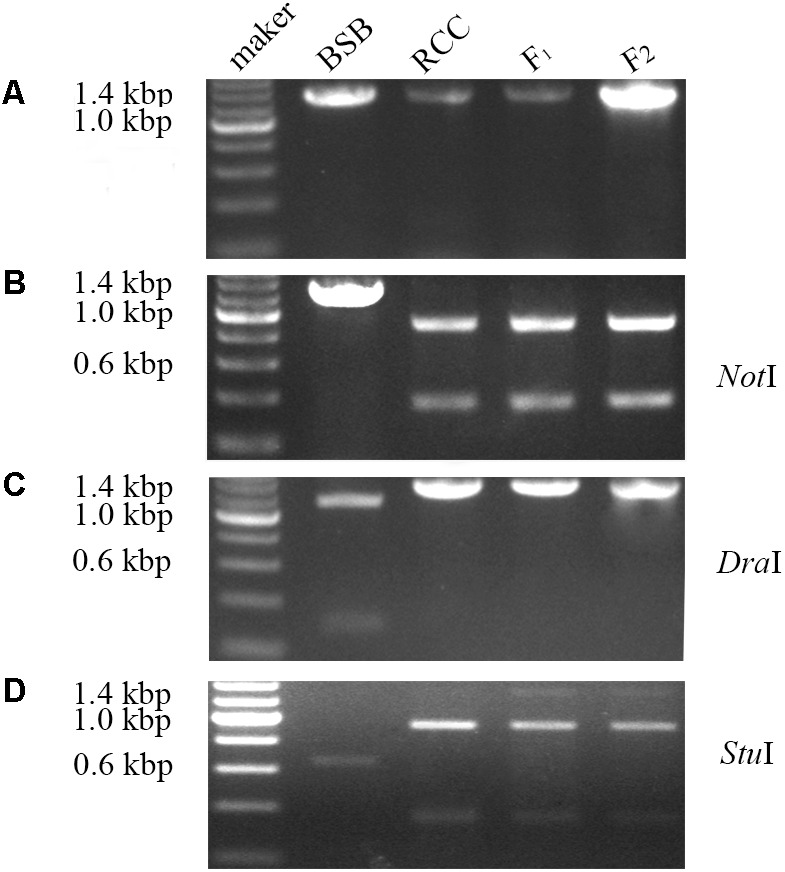
Analysis of the inheritance pattern of complete *ITS* region in distant hybrid lineage of RCC × BSB. **(A)** The complete DNA sequence of *ITS* from distant hybrid lineage of RCC × BSB without digestion by enzyme. **(B)** The digested product by *Not*I from distant hybrid lineage of RCC × BSB. **(C)** The digested product by *Dra*I from distant hybrid lineage of RCC × BSB. **(D)** The digested product by *Stu*I from distant hybrid lineage of RCC × BSB.

Internal transcribed spacer sequence polymorphisms allow distinct nucleolar organizing regions (NORs) to be identified via specific restriction enzyme sites in parental individuals and polyploid hybrid progeny (**Figure 2**). Lengths of DNA fragments (including partial *18S rDNA*, complete *ITS* regions, and partial *28S rDNA* sequences) were amplified from the BSB (1296 bp) (Accession No. MG830472) and RCC (1283 bp) (Accession No. MG830471). In the F_1_ and F_2_ hybrids, the length of the *ITS* region was 1283 bp (**Figure 2A**). The *Not*I restriction enzyme could digest the *ITS* region into two smaller bands in the RCC (904 and 379 bp), and in the F_1_ (904 and 379 bp) and F_2_ (904 and 379 bp) hybrids, but not in the BSB (**Figure 2B**). The *Dra*I restriction site was used because it was found in the *ITS* region of BSB (1039 bp), but not in the *ITS* region of RCC, or of the F_1_ or F_2_ hybrids (**Figure 2C**). The *Stu*I restriction site could be found in the *ITS* region of both BSB (658 bp) and RCC (926 bp). In the F_1_ and F_2_ hybrids only a proportion of the individuals carried the *Stu*I restriction site, although where it was present was in the same position as in the RCC (**Figure 2D**). (Note: Three samples showed the same results.)

### Expression Pattern of the *ITS* Sequence in Parental Species and Hybrids

**FIGURE 3:**
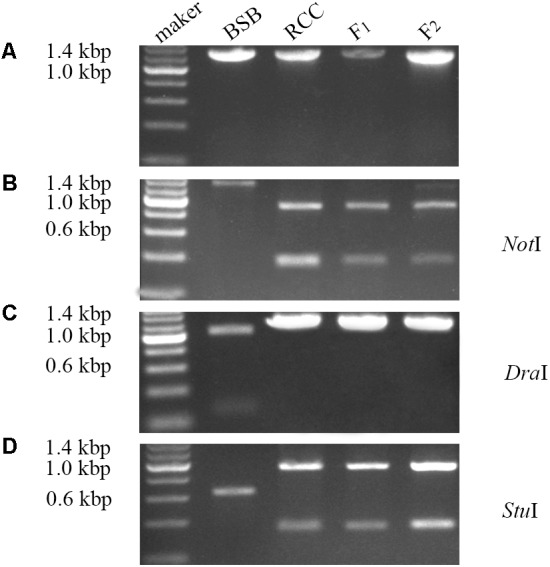
Analysis of the expression pattern of complete *ITS* region in distant hybrid lineage of RCC × BSB. **(A)** The complete cDNA sequence of *ITS* from distant hybrid lineage of RCC × BSB without digestion by enzyme. **(B)** The digested product by *Not*I from distant hybrid lineage of RCC × BSB. **(C)** The digested product by *Dra*I from distant hybrid lineage of RCC × BSB. **(D)** The digested product by *Stu*I from distant hybrid lineage of RCC × BSB.

Expression of the complete *ITS* sequence was amplified in BSB (1296 bp), RCC (1283 bp), and the F_1_ (1283 bp) and F_2_ (1283 bp) hybrids (**Figure 3A**). A *Not*I restriction site was found in the cDNA fragment of the *ITS* region in RCC (904 bp), but not in BSB. Both the F_1_ and F_2_ hybrids carried a restriction site in the same position as RCC, although in some of the F_2_ hybrids the position of this restriction site varied (**Figure 3B**). A *Dra*I restriction site was found in the cDNA fragment of the *ITS* region in BSB (1039 bp), but not in RCC, or the F_1_ and F_2_ hybrids (**Figure 3C**). A *Stu*I restriction site was found in the cDNA fragment of the *ITS* regions of both BSB (658 bp), RCC (926 bp), the F_1_ (926 bp) and F_2_ (926 bp) hybrids (**Figure 3D**). (Note: Three samples showed the same results.)

### Sequence and Variation of *18S rRNA* Genes in Parental Species

We examined the intra- and interspecific sequence divergence for 60 sequences from 30 RCC individuals and 60 sequences from 30 BSB individuals. The sequence of the *18S rRNA* gene in RCC was 1558 bp (Accession No. MG830470). In BSB, the sequence of the *18S rRNA* gene was homogeneous with the sequence provided by Xiao et al. (2016) (Accession No. AB860215), except for position 12 (indicated with asterisk in **Supplementary Figure S1**). The similarity between *18S rRNA* gene fragments amplified from RCC and BSB genome was 97.00% (**Table 2** and **Supplementary Figure S1**).

**Table 2:** The similarity comparisons of the DNA of *18S rRNA* gene in RCC, BSB, F_1_ and F_2_ hybrids.

Similarity		RCC	BSB	F_1_		F_2_	
				Pattern 1	Pattern 2	Pattern 1	Pattern 2
RCC			97%	100%	99%	100%	99%
BSB				97%	97%	97%	97%
F_1_	Pattern 1				99%	100%	99%
	Pattern 2					99%	100%
F_2_	Pattern 1						99%
	Pattern 2						

*RCC, Carassius auratus red var.; BSB, Megalobrama amblycephala; F_1_, allotetraploids; F_2_, autotetraploids.*

### Expression of the *45S rRNA* Gene

**FIGURE 4:**
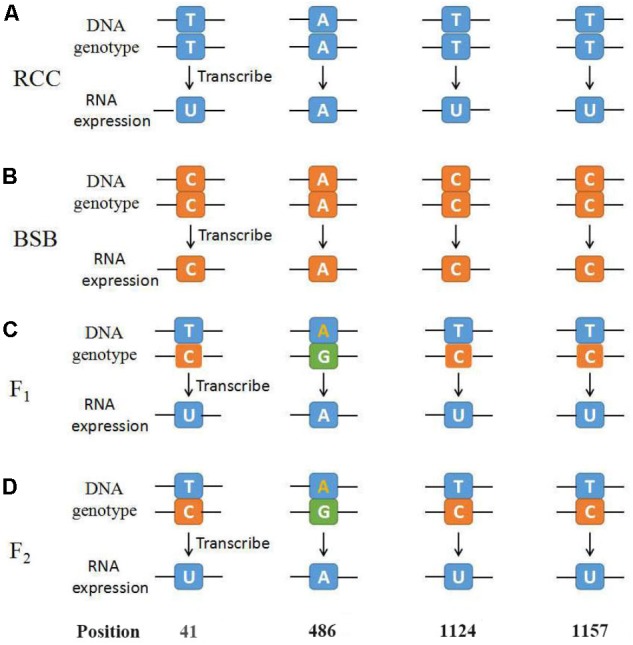
The diagrammatic drawing of genotype and expression of *45S rRNA* gene at position 41, 486, 1124, and 1157. **(A)** The genotype and expression of *45S rRNA* gene in RCC. **(B)** The genotype and expression of *45S rRNA* gene in BSB. **(C)** The genotype and expression of *45S rRNA* gene in F_1_ hybrids of RCC × BSB. **(D)** The genotype and expression of *45S rRNA* gene in F_2_ hybrids of RCC × BSB. For every pattern of each kind of fish, one sample was used to present.

Thirty samples each of fish (RCC, BSB, F_1_ and F_2_ hybrids) were selected. According to genotypes of *18S rRNA* gene, the inheritance pattern in hybrids fell into two classes. In pattern 1, the DNA sequence of the 18S *rRNA* gene was consistent with that of RCC. In pattern 2, the DNA sequence of the 18S *rRNA* gene had variations in four positions compared with that of RCC: position 41 (T→C), 486 (A→G), 1124 (T→C), and 1157 (T→C) (**Figure 4** and **Supplementary Figure S1**). The proportion of F_1_ hybrids belonging to pattern 1 was 76.67% and the proportion belonging to pattern 2 was 23.33% (**Table 3**). The proportion of F_2_ hybrids belonging to pattern 1 was 73.33% and the proportion belonging to pattern 2 was 26.67% (**Table 4**). The four positions 41, 486, 1124, and 1157 in the F_1_ and F_2_ hybrids were examined by primers (**Supplementary Table S1** and **Supplementary File S1**). The cDNAs of the *18S rRNA* gene in all the F_1_ and F_2_ hybrids were consistent with that of RCC, whether they belonged to pattern 1 or 2 (**Figure 4**).

**Table 3:** The frequency distribution of the 2 different genetic expression patterns of *45S rRNA* gene in the F_1_ hybrids.

	Number	Percent
Pattern 1^a^	23	76.67
Pattern 2^a^	7	23.33
Total	30	100

*^a^Pattern 1 inherits the single 45S rRNA gene of RCC, Pattern 2 inherits the single 45S rRNA gene of RCC but it occurs nucleotide variation. Pattern 1 and Pattern 2 express the single 45S rRNA gene of RCC.*

**Table 4:** The frequency distribution of the two different genetic expression patterns of *45S rRNA* gene in the F_2_ hybrids.

	Number	Percent
Pattern 1^a^	22	73.33
Pattern 2^a^	8	26.67
Total	30	100

^*a*^Pattern 1 inherits the single 4*5S rRNA* gene of RCC, Pattern 2 inherits the single *45S rRNA* gene of RCC but it occurs nucleotide variation. Pattern 1 and Pattern 2 express the single *45S rRNA* gene of RCC.

## Discussion

The *45S rRNA* gene is a classic locus for the study of both genomic structure and expression levels in polyploids, as part of a study of the more general topic of nucleolar dominance. The knowledge of understanding the nucleolar dominance in hybrid origin’s fish and polyploidy livestock is scarce. The limited data indicate that nucleolar dominance have established in the red vizcacha rat (Gallardo et al., 2006). However, the nucleolar dominance established in a hybrids fish lineage is not realized until the second generation (Xiao et al., 2016). In the present study, we observed that in the F_1_ hybrids of RCC × BSB, the inherited and expressed *45S rRNA* genes were derived from the females, RCC. Thus, preferential expression evolved rapidly in the initial generations. The F_1_ hybrids can successfully survive in the first generation of RCC × BSB, but diploids hybrids cannot, probably because of the incompatibility of paternal genome (Qin et al., 2015). Polyploidization-associated genomic changes have been found in F_1_ hybrids (Qin et al., 2016). Thus, we speculate that tetraploidization facilitate rapid establishment of nucleolar dominance in F_1_ hybrids. As reported, genetics changes in the hybrids may have some relationship with the unique nucleolar dominance patterns in the hybrid lineage (Xiao et al., 2016). In Xenopus hybrids, nucleolar dominance appears to be associated with a major non-Mendelian reduction in the number of 45S *rRNA* gene copies rather than a specific pattern of their expression. The loss of *rRNA* gene copies in F_1_ hybrids was non-random with respect to the parental species, with the transcriptionally dominant variant preferentially removed from hybrid zygotes (Katarzyna et al., 2015). The “epigenetic landscape” created by cytosine methylation and various histone modification also can play an important role in selective silencing of *rRNA* genes (Chen and Pikaard, 1997; Olga et al., 2007). The specific molecular basis for choosing *45S rRNA* genes of RCC to express in F_1_ hybrids still needs further exploration.

Four single nucleotide variations were observed in the DNA sequences of *18S rRNA* gene of F_1_ hybrids. Incomplete homogenization of *18S* ribosomal DNA sequences have been reported in some studies (Mentewab et al., 2011; Chelomina et al., 2016). Polyploidy and hybridization are the most often discussed reasons for nucleotide variability in the *rDNA* sequences (Muir et al., 2001; Liu et al., 2003). Thus, tetraploidization and distant hybridization may be responsible for the appearance of this nucleotide variation. Exosome mediated quality control of misfolded pre-*rRNAs* following their polyadenylation (Kadaba et al., 2006; Slomovic et al., 2006; Chekanova et al., 2007) as well as the ‘non-functional *rRNA* decay’ leading to decreased stability of the mature *rRNA* contained in fully assembled ribosomes and ribosomal subunits (Lariviere et al., 2006). These mechanisms can explain that cDNAs of the *18S rRNA* gene in all the F_1_ and F_2_ hybrids were consistent with that of RCC, whether they belonged to pattern 1 or 2. It was further confirmed by the digestion products of the restriction enzyme in the complete *ITS* region. In addition, we found an unrecognized band due to the *Not*I enzyme in the cDNA of the complete *ITS* of F_2_ hybrids. Because the *5.8S* region in all parents and hybrids was conservation, the variant restriction enzyme site existed in *ITS* region. The F_2_ hybrids were produced through the elimination of BSB genetic material and an autopolyploidization process. It is reasonable to assume that these genomic changes cause variation of the cDNA of the *ITS* region in the F_2_ hybrids.

Nucleolar dominance from the females was established in the F_1_ hybrids and it was inherited in the F_2_ hybrids, suggesting that tetraploidization can lead to rapid establishment of nucleolar dominance in the hybrid origin’s tetraploid lineage. These results extend the knowledge of nucleolar dominance in polyploidy hybrid animals, which are of significance for the evolution of hybrids in vertebrates.

## Ethics Statement

All the fish were cultured in ponds at the Protection Station of Polyploid Fish, Hunan Normal University. According to the Care and Use of Agricultural Animals in Agricultural Research and Teaching, fish treatments were performed. These were approved through the Science and Technology Bureau of China. Approval from the Department of Wildlife Administration was not required for the experiments conducted in this study. Before dissection, fish were deeply anesthetized with 100 mg/L MS-222 (Sigma-Aldrich).

## Author Contributions

SL contributed to the conception and designed the study. LC, QQ, QX, HY, and JW carried out the experimental work and participated in drafting the manuscript. QL, XH, and YH analyzed the sequences. MT, CZ, and KL participated in interpretation and discussion of the results. All authors read and approved the final manuscript.

## Conflict of Interest Statement

The authors declare that the research was conducted in the absence of any commercial or financial relationships that could be construed as a potential conflict of interest.

## Supplementary Material

FIGURE S1 Sequence alignment of 18S *rDNA* of pattern 2 in RCC, BSB, F_1_ and F_2_ hybrids. Variable sites are shaded; asterisks mark position 12.

TABLE S1 The primers are designed to examine the position 41, 486, 1124, and 1157 in F_1_ and F_2_ hybrids.

FILE S1 The sequencing peak figures of the PCR products amplified from genomic of 18S rRNA gene. Only the position 41 and 2 upstream and downstream nucleotides are showed. **(B)** The sequencing peak figures of the PCR products amplified from genomic of 18S rRNA gene. Only the position 486 and 2 upstream and downstream nucleotides are showed. **(C)** The sequencing peak figures of the PCR products amplified from genomic of 18S rRNA gene. Only the position 1124 and 2 upstream and downstream nucleotides are showed. **(D)** The sequencing peak figures of the PCR products amplified from genomic of 18S rRNA gene. Only the position 1157 and 2 upstream and downstream nucleotides are showed.
