# Supplementary figures and images for: Nucleolar Dominance in a Tetraploidy Hybrid Lineage Derived From Carassius auratus red var. ([image]) × Megalobrama amblycephala ([image])
Source: Front Genet. 2018 Sep 24;9:386. doi: 10.3389/fgene.2018.00386 (PMC6166360; doi:10.3389/fgene.2018.00386)

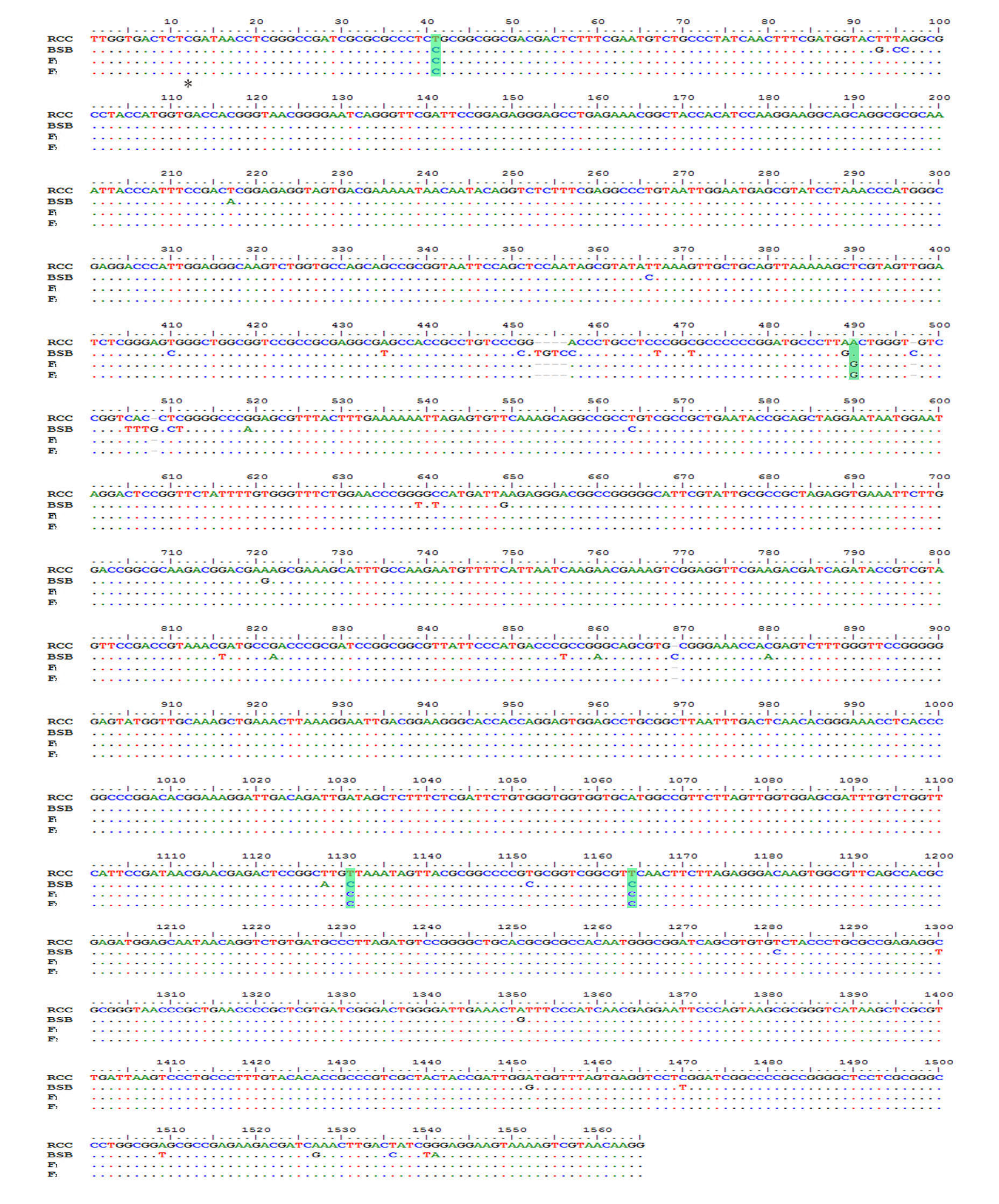

Supplement: FIGURE S1 — Sequence alignment of 18S rDNA of pattern 2 in RCC, BSB, F1 and F2 hybrids. Variable sites are shaded; asterisks mark position 12. [file Image_1.jpg]

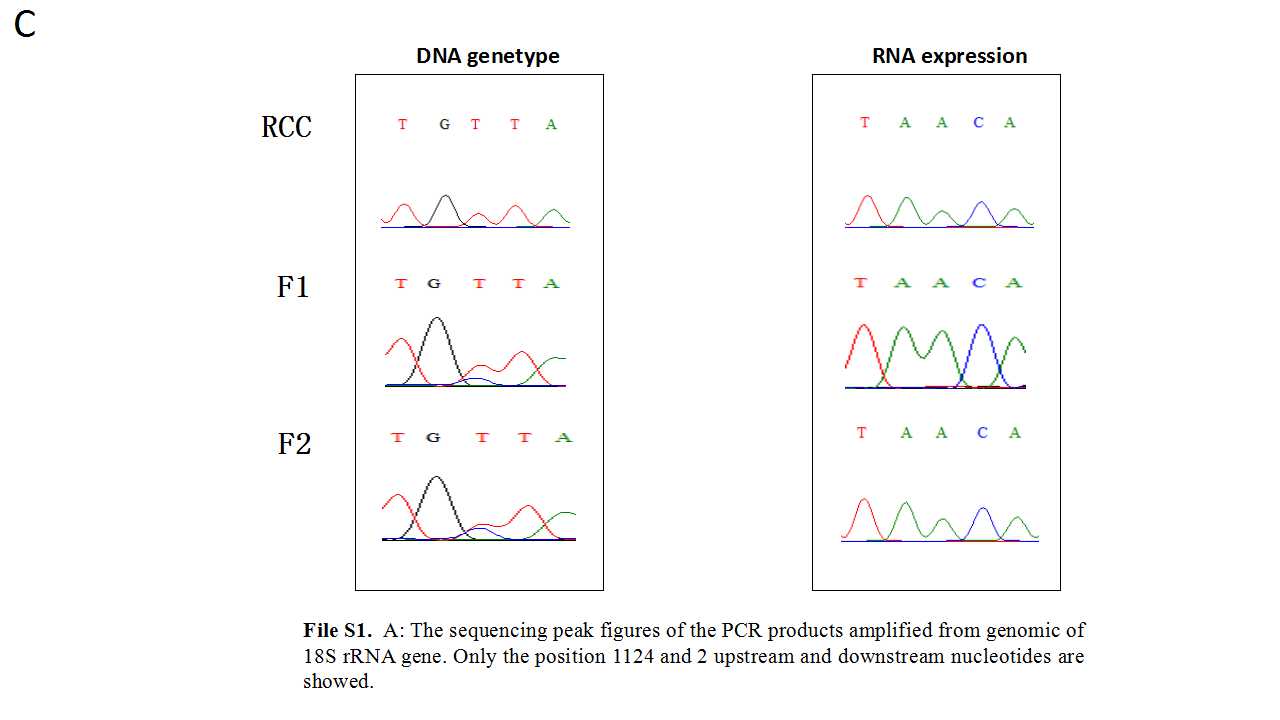

Supplement: FILE S1 — The sequencing peak figures of the PCR products amplified from genomic of 18S rRNA gene. Only the position 41 and 2 upstream and downstream nucleotides are showed. (B) The sequencing peak figures of the PCR products amplified from genomic of 18S rRNA gene. Only the position 486 and 2 upstream and downstream nucleotides are showed. (C) The sequencing peak figures of the PCR products amplified from genomic of 18S rRNA gene. Only the position 1124 and 2 upstream and downstream nucleotides are showed. (D) The sequencing peak figures of the PCR products amplified from genomic of 18S rRNA gene. Only the position 1157 and 2 upstream and downstream nucleotides are showed. [file Presentation_1.ZIP › File S1/position1124.jpg]

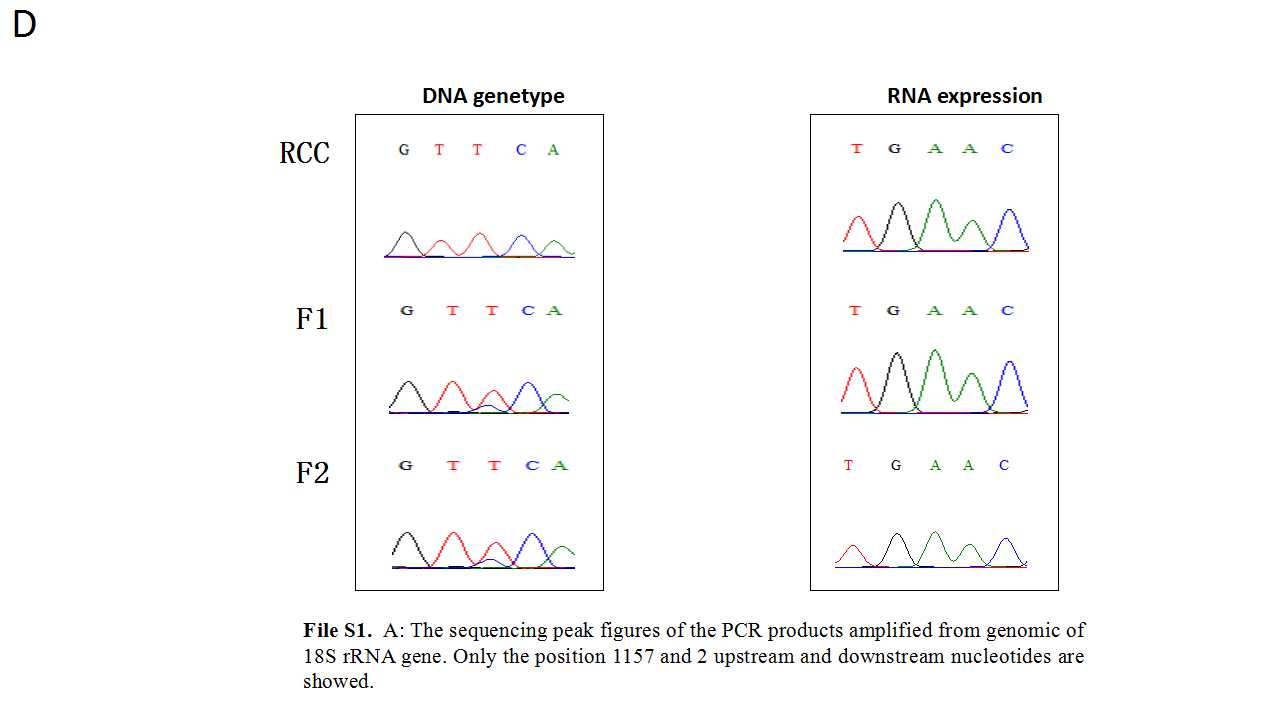

Supplement: FILE S1 — The sequencing peak figures of the PCR products amplified from genomic of 18S rRNA gene. Only the position 41 and 2 upstream and downstream nucleotides are showed. (B) The sequencing peak figures of the PCR products amplified from genomic of 18S rRNA gene. Only the position 486 and 2 upstream and downstream nucleotides are showed. (C) The sequencing peak figures of the PCR products amplified from genomic of 18S rRNA gene. Only the position 1124 and 2 upstream and downstream nucleotides are showed. (D) The sequencing peak figures of the PCR products amplified from genomic of 18S rRNA gene. Only the position 1157 and 2 upstream and downstream nucleotides are showed. [file Presentation_1.ZIP › File S1/position1157.jpg]

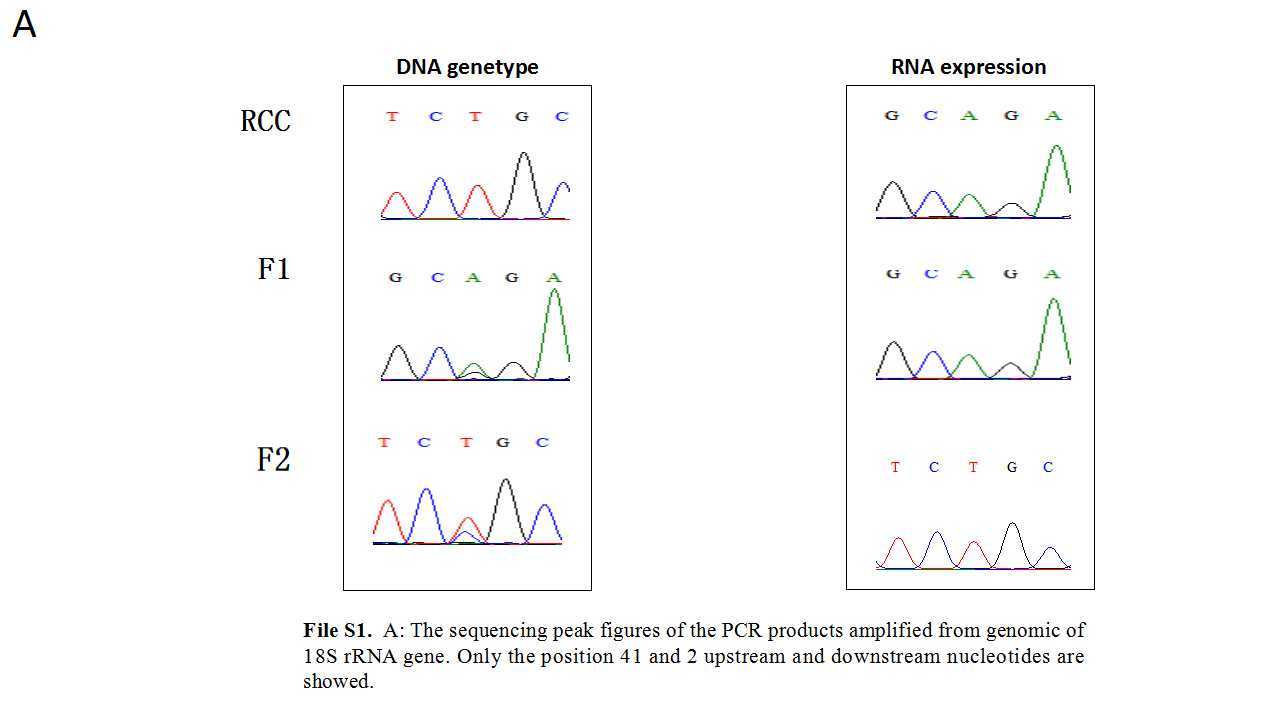

Supplement: FILE S1 — The sequencing peak figures of the PCR products amplified from genomic of 18S rRNA gene. Only the position 41 and 2 upstream and downstream nucleotides are showed. (B) The sequencing peak figures of the PCR products amplified from genomic of 18S rRNA gene. Only the position 486 and 2 upstream and downstream nucleotides are showed. (C) The sequencing peak figures of the PCR products amplified from genomic of 18S rRNA gene. Only the position 1124 and 2 upstream and downstream nucleotides are showed. (D) The sequencing peak figures of the PCR products amplified from genomic of 18S rRNA gene. Only the position 1157 and 2 upstream and downstream nucleotides are showed. [file Presentation_1.ZIP › File S1/position41.jpg]

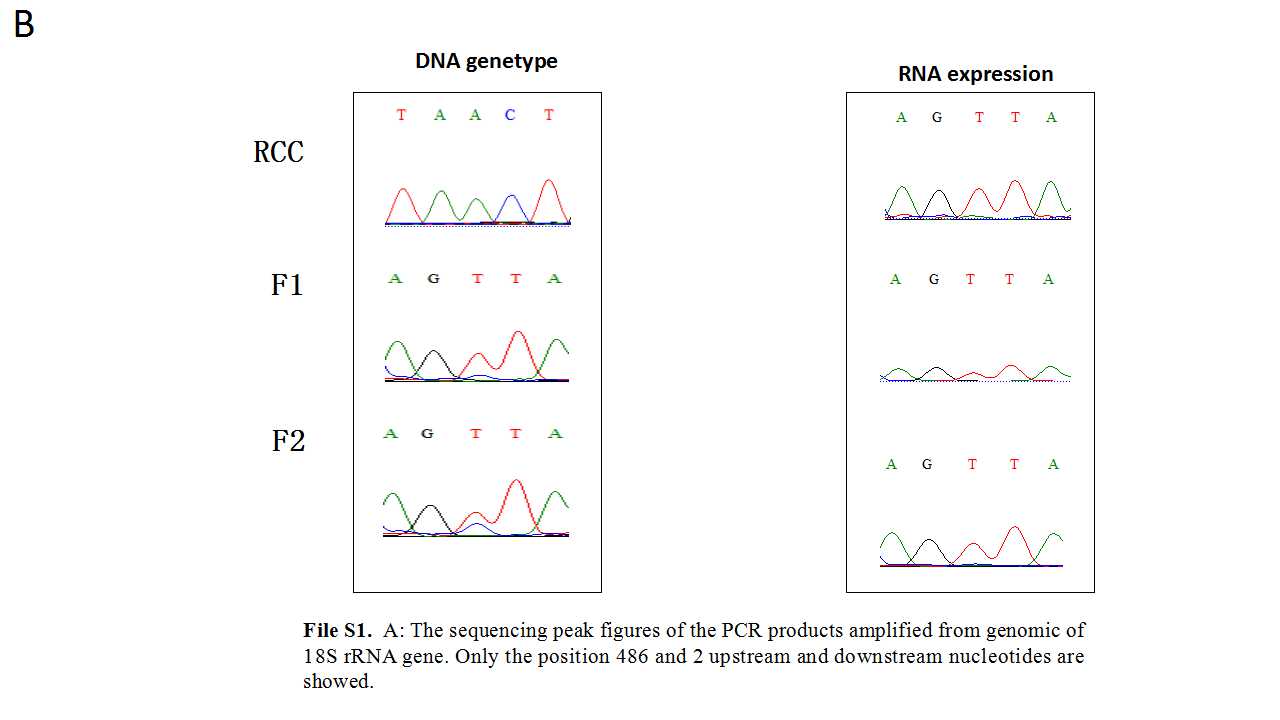

Supplement: FILE S1 — The sequencing peak figures of the PCR products amplified from genomic of 18S rRNA gene. Only the position 41 and 2 upstream and downstream nucleotides are showed. (B) The sequencing peak figures of the PCR products amplified from genomic of 18S rRNA gene. Only the position 486 and 2 upstream and downstream nucleotides are showed. (C) The sequencing peak figures of the PCR products amplified from genomic of 18S rRNA gene. Only the position 1124 and 2 upstream and downstream nucleotides are showed. (D) The sequencing peak figures of the PCR products amplified from genomic of 18S rRNA gene. Only the position 1157 and 2 upstream and downstream nucleotides are showed. [file Presentation_1.ZIP › File S1/position486.jpg]
