# Supplementary material for: Nucleolar Dominance in a Tetraploidy Hybrid Lineage Derived From Carassius auratus red var. ([image]) × Megalobrama amblycephala ([image])
Source: Front Genet. 2018 Sep 24;9:386. doi: 10.3389/fgene.2018.00386 (PMC6166360; doi:10.3389/fgene.2018.00386)
Supplement: TABLE S1 — The primers are designed to examine the position 41, 486, 1124, and 1157 in F1 and F2 hybrids. [file Table_1.DOCX]

**Table S1 The primers are designed to examine the position 41, 486, 1124 and 1157 in F_1_ and F_2_** **hybrids**

| Position | Primers |
| --- | --- |
| 41 | F:GATTAAGCCATGCAGGTCTAAGTG  R:TTGGATGTGGTAGCCGTTTCT |
| 486 | F:CTCGTAGTTGGATCTCGGGAGTG  R:GGCGGCCTGCTTTGA  ACACT |
| 1124 and 1157 | F:CTTTCTCGATTCTGTGGGTGG  R:CGGACATCTAAGGGCATCAC |
